# Does Systemic Hematological Therapy Influence the Course of Paraproteinemic Keratopathy?

**DOI:** 10.3390/jcm13020565

**Published:** 2024-01-18

**Authors:** Mohammad Al Hariri, Markus Munder, Norbert Pfeiffer, Joanna Wasielica-Poslednik

**Affiliations:** 1 Department of Ophthalmology, University Medical Center of the Johannes Gutenberg-University Mainz, 55131 Mainz, Germany; norbert.pfeiffer@unimedizin-mainz.de (N.P.); joanna.wasielica-poslednik@unimedizin-mainz.de (J.W.-P.); 2 Department of Hematology and Oncology, University Medical Center of the Johannes Gutenberg-University Mainz, 55131 Mainz, Germany; markus.munder@unimedizin-mainz.de

**Keywords:** paraproteinemic keratopathy, monoclonal gammopathy of ocular significance, multiple myeloma, in vivo confocal laser scanning microscopy, keratoplasty

## Abstract

The purpose of this article is to evaluate the course of paraproteinemic keratopathy (PPK) in patients undergoing systemic therapy for the underlying hematological disease. Baseline and follow-up examinations included hematological work-up, best-corrected visual acuity, slit-lamp biomicroscopy, and in vivo confocal laser scanning microscopy (IVCM). We included 22 patients with bilateral PPK (aged 68 ± 10.4 years, 11 males). Ten patients with multiple myeloma (MM) underwent on-label systemic therapy. During follow-up, we observed a regression of corneal opacities in three patients under slit-lamp examination and under IVCM, while PPK remained unchanged in seven patients. In three patients with monoclonal gammopathy of ocular significance (MGOS), systemic therapy was initiated off-label to reduce the serum paraprotein load before penetrating keratoplasty (PKP). These patients showed no signs of PPK recurrence for up to 24 months after PKP. In one patient without systemic therapy, a recurrence in corneal grafts occurred within 12 months of PKP. In eight patients without systemic therapy, PPK remained stable. In conclusion, systemic therapy for MM patients reduced corneal opacity in 30% of treated patients. Furthermore, systemic therapy performed before PKP in patients without conventional systemic therapy indication (MGOS) likely postpones PPK recurrence in the corneal graft.

## 1. Introduction

Paraproteinemic keratopathy (PPK) is a corneal condition potentially affecting patients with monoclonal gammopathy (MG). This family of lymphoproliferative disorders includes multiple myeloma (MM) and its precancerous stages, smoldering multiple myeloma (SMM) and monoclonal gammopathy of unknown significance (MGUS) [1]. MG is caused by the secretion of monoclonal immunoglobulins or light chains only (so called paraprotein or M-protein), via an expanded clone of plasma cells within the bone marrow [1]. Deposition of paraprotein in the cornea may result in crystalline or non-crystalline corneal opacities of a very heterogenic morphology. According to our previous study, PPK is present in about one-third of MG patients, mostly in the form of discreet corneal stromal opacities, which do not relevantly influence visual acuity, but in rare cases may cause a relevant visual impairment [2]. Ocular diseases associated with MG, such as corneal or macular involvement, without evidence of other organ damage due to the underlying MG, have recently been defined as “monoclonal gammopathy of ocular significance” (MGOS) [3]. According to our observations, the severity of PPK does not correlate with the severity of the underlying disease or the concentration of the M-protein in serum. We observed severe corneal opacities in patients with MGUS and low paraprotein levels, and on the other hand no or mild PPK in patients with MM [2].

In the context of MG, different organs can be affected by the deposition of paraproteins. The manifestation of MGOS varies between PPK, corneal copper aggregation, paraproteinemic maculopathy, and retinal vascular occlusion [4,5]. Less commonly, paraprotein deposition was seen in the conjunctiva, lens, ciliary body, pars plana, choroid, and orbit [5,6,7,8,9].

The mechanism of paraprotein deposition in the cornea is largely unclear. It has been hypothesized that the depth of the deposits can be used to infer the source of the deposited material, for example, whether it enters the cornea from the tear film, limbal vessels, or aqueous humor [10].

In vivo laser scanning confocal microscopy (IVCM) is an important examination tool for diagnosing PPK, allowing visualization of paraprotein deposits (punctiform or needle-like) in the corneal stroma [2,5,11,12].

An explicit treatment of PPK is not available so far. According to hematological guidelines, PPK, or even MGOS, are not considered as end-organ damaging, and even severe visual impairment is not an indication so far for an in-label systemic treatment [13]. Systemic therapy is applied to patients fulfilling CRAB criteria (hyper**c**alcemia, **r**enal failure, **a**nemia, or lytic **b**one lesions), or when certain biomarkers are present (clonal bone marrow plasma cells ≥ 60%, serum free light-chain (FLC) ratio ≥ 100 (provided the involved FLC level is ≥100 mg/L), or more than one focal lesion on an MRI) [14].

There are some case reports describing an “accidental” reduction of PPK under systemic treatment [11,15]. So far, there are very few reports regarding the application of systemic treatment to patients suffering from clinically relevant PPK but not meeting CRAB criteria (e.g., MGUS) [4]. Penetrating keratoplasty (PKP) is the only treatment option in the case of massive loss of visual function following PPK. However, the paraprotein deposits recur in most cases within the first 12 months after PKP [11,16,17,18,19,20,21,22,23,24]. It is unclear if systemic treatment prior to PKP may postpone or prevent PPK recurrence in the graft [11,16,17,18,23,24].

In our study, we followed patients with a diagnosis of PPK, with and without systemic treatment, for several years. We aimed to find out whether systemic therapy influences the course of PPK. The clinical decisions regarding therapy were interdisciplinary, made by hematologists and ophthalmologists. We hypothesized that systemic plasma cell dyscrasia-directed therapy (with or without pure hematological indication) may postpone or prevent PPK recurrence in the corneal graft.

## 2. Materials and Methods

This prospective observational cross-sectional clinical study was conducted between April 2016 and March 2023 to investigate the course of PPK in patients undergoing systemic therapy of the underlying MG. We published the results of the prevalence in 2022 [2]. Moreover, we recruited and controlled additional patients who had confirmed PPK, some of whom were treated systemically and/or surgically. This study is registered and has ethics approval from the Ethics Commission of Rhineland-Palatinate, Germany [vote no. 837.153.16 (10472)]. All study participants provided written informed consent and were examined at the Department of Ophthalmology and at the Department of Hematology and Oncology of the University Medical Center of the Johannes Gutenberg University Mainz.

In this study we present an ophthalmological and hematological follow-up of 1.5–7 years for 22 patients identified with the diagnosis of bilateral PPK. In ten patients, the underlying hematological disease was MM, which was treated by the hematologists according to guidelines [14]. Twelve patients suffering from PPK due to non-MM plasma cell dyscrasia (MGUS, SMM) or lymphoma (lymphoplasmacytoid immunocytoma) did not require systemic treatment from a hematologic perspective.

### 2.1. Hematological Evaluation

Hematological examination, diagnosis, treatment, and post-therapeutic monitoring were performed according to the current guidelines of the Department of Hematology and Oncology, University Medical Center of the Johannes Gutenberg University Mainz [25]. These included, among other things, immunofixation in serum, immunoglobulins (Ig) G, A, M, D, and E, kappa (κ)—and lambda (λ) light-chains (LC) in serum, kappa/lambda ratios, free kappa light chains (FLC κ), free lambda light chains (FLC λ), free kappa/lambda light-chain ratios, and M-protein and serum protein electrophoresis. Further investigations were conducted such as low-dose whole-body computed tomography (CT), whole-body magnetic resonance imaging (MRI), whole-body positron emission tomography/computed tomography (PET/CT), and histopathological examination of a bone marrow specimen.

### 2.2. Ophthalmological Evaluation

The ophthalmological evaluation at baseline as well as at follow-up included: an autorefractometer (model AR-360A, Nidek Co, Wetzlar, Germany), best-corrected visual acuity (BCVA) measured with Snellen charts; slit lamp biomicroscopy, fundoscopy, optical coherence tomography (OCT, Spectralis: anterior segment module and macular volume scan, Heidelberg Engineering, Heidelberg, Germany) of the cornea and macula, Goldmann applanation tonometry and IVCM (HRT2-04318 Rostock Cornea Model, Heidelberg Engineering GmbH, Heidelberg, Germany) [2].

The regression of PPK was defined as a reduction or disappearance of corneal opacities under a slit lamp and a reduction or disappearance of corneal paraprotein deposits under IVCM examination. Absence of PPK recurrence in the graft was defined as clear graft without PPK-like findings under biomicroscopy and IVCM. The regression of PPK was an objective finding based on the slit lamp examination, including slit lamp photos and IVCM performed by one observer and evaluated by two observers, with the second observer assessing the ophthalmological findings without information about any systemic therapy.

## 3. Results

We included 22 patients with bilateral PPK associated with an underlying hematological disease (MM, MGUS, or lymphoma). All patients had bilateral corneal opacity under slit lamp and needle-like deposits in the corneal stroma under IVCM, confirming the association with MG. Table 1 presents demographic data as well as systemic diagnoses of the patients.

### 3.1. Patients Requiring Systemic Treatment according to CRAB Criteria

**Figure 1:**
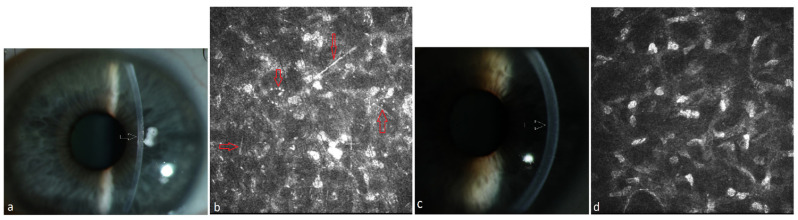
Patient with multiple myeloma of type IgA lambda (#8, Table 2). (**a**) Diffuse stromal flake-like PPK (white arrow) in the left eye before systemic therapy, (**b**) needle-like and punctiform deposits (red arrows) in the stroma of the left eye at a depth of 219 µm under IVCM, (**c**) clear cornea (white arrow = absence of the opacity) of the left eye due to recovery from PPK after systemic therapy and autoSCT, (**d**) absence of paraprotein deposits in the stroma at a depth of 241 µm after therapy.

**Figure 2:**
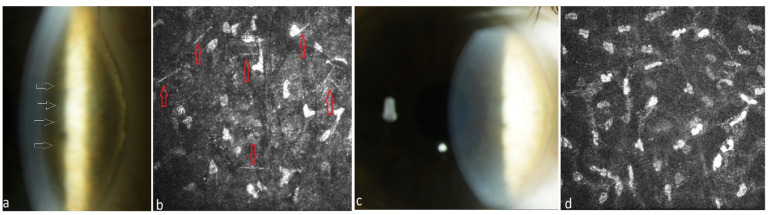
Patient with multiple myeloma of type IgG kappa (#9, Table 2). (**a**) Stromal punctiform crystalline-like PPK (white arrows) in the right eye before systemic therapy, (**b**) needle-like deposits (red arrows) in the stroma of the right eye at a depth of 186 µm under IVCM, (**c**) clear cornea of the right eye after systemic plasma cell directed therapy and autoSCT, (**d**) absence of paraprotein deposits in the stroma at a depth of 194 µm after therapy.

**Figure 3:**
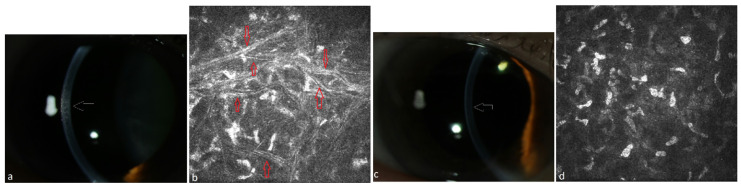
Patient with multiple myeloma of type IgA lambda (#10, Table 2). (**a**) Stromal flake-like opacity (white arrow) in the right eye before systemic therapy, (**b**) needle-like deposits (red arrows) in the stroma of the right eye at a depth of 152 µm under IVCM, (**c**) clear cornea of the right eye (white arrow = absence of the opacity) due to recovery from PPK after systemic therapy, (**d**) absence of paraprotein deposits in the stroma at a depth of 150 µm after therapy.

Ten patients suffering from MM meeting CRAB criteria received systemic therapy of the underlying disease. The ophthalmological and hematological follow-up of patients under systemic therapy is shown in Table 2. These patients had not received any local ophthalmological therapy. All these patients presented a mild PPK at baseline and had not required any surgical treatment. The follow-up of three patients was lost after 13–33 months due to death. Until that time, the PPK remained stable in these patients (#1, #2, #3 in Table 2). The follow-up of seven patients for up to 4–8 years after or during systemic treatment showed a regression of PPK in three cases (#8, #9, #10 in Table 2, Figure 1, Figure 2 and Figure 3). One patient presented additionally paraproteinemic maculopathy (PPM) in the form of focal detachment of the neurosensory retina with secondary choroidal neovascularization (CNV) in the right eye (#10 in Table 2).

### 3.2. Patients Not Requiring Systemic Treatment According to CRAB Criteria

**Figure 4:**
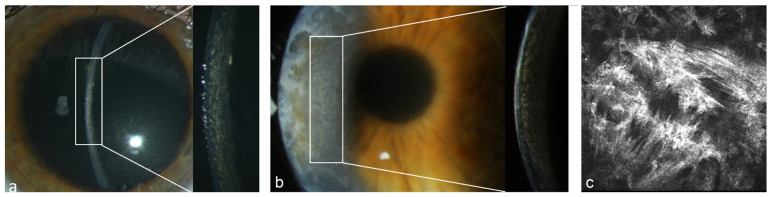
Patient with MGUS of type IgG kappa (#1 Table 3). (**a**) Stromal flake-like PPK in the right eye, (**b**) recurrence of stromal flake-like PPK in the right eye 12 months after PKP, (**c**) hyperreflective needle-like deposits in the stroma of the right eye at a depth of 449 µm after PKP under IVCM.

**Figure 5:**
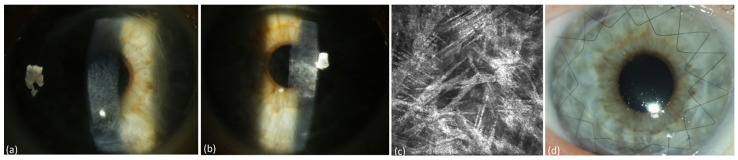
Patient with systemic light-chain (AL) amyloidosis type lambda (#2 Table 3). (**a**) Deep stromal crystalline PPK of the right eye, (**b**) deep stromal crystalline PPK of the left eye in a patient, (**c**) hyperreflective needle-like deposits in the stroma of the right eye at a depth of 413 µm under IVCM, (**d**) recurrence-free corneal graft of the right eye after surgery.

**Figure 6:**
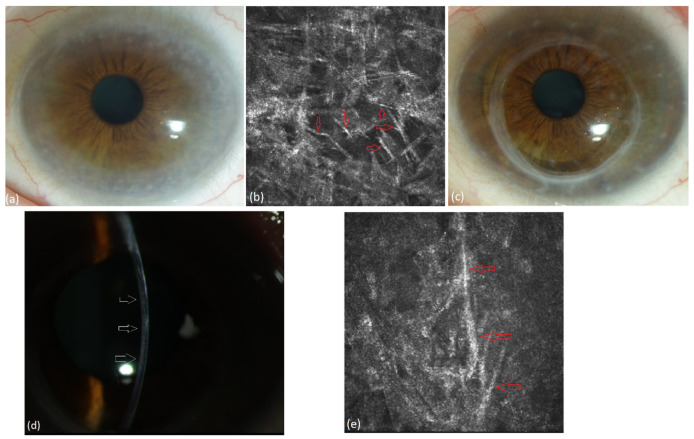
Patient with lymphoplasmacytoid immunocytoma and MGUS of type IgG kappa (#3, Table 3). (**a**) Diffuse stromal flake-like opacity in the left eye preoperatively, (**b**) extracellular needle-like deposits (red arrows) in the stroma of the left eye at a depth of 503 µm under IVCM, (**c**) recurrence-free corneal graft 24 months after surgery, (**d**) mild stromal recurrence of PPK (wihte arrows) after 27 months, (**e**) extracellular needle-like deposits (red arrows) in the stroma of the left eye at a depth of 538 µm 27 months after PKP.

**Figure 7:**
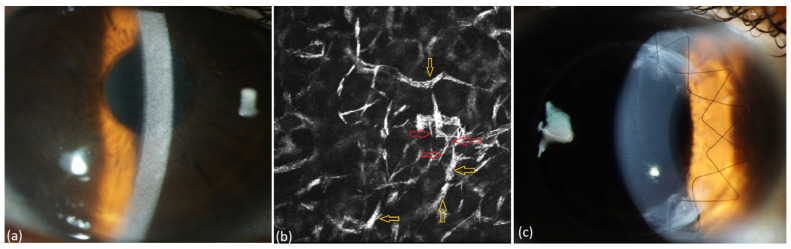
Patient with MGUS of type IgG kappa (#4, Table 3). (**a**) Diffuse stromal flake-like opacity in the right eye, (**b**) intracellular (yellow arrow) and extracellular (red arrow) needle-like deposits in the stroma of the right eye at a depth of 494 µm preoperatively, (**c**) recurrence-free corneal findings one year after surgery.

We present the follow-up of the 12 untreated patients in Table 3. Four out of twelve patients presented severe bilateral PPK affecting visual acuity at baseline. One patient got bilateral PKP before the diagnoses of MGUS and PPK were made (#1 in Table 3, Figure 4) and PPK recurred within 12 months after PKP. His case is described elsewhere [16]. Three patients (#2, #3 and #4 in Table 3, Figure 5, Figure 6 and Figure 7) received off-label systemic therapy to reduce paraprotein concentration in serum before PKP to reduce the risk of a quick recurrence in the graft. We performed PKP 9–12 months after the start of systemic treatment, as soon as the M-Protein was significantly reduced. Eight patients did not require any surgical intervention. In seven patients, the corneal opacity stayed stable in the follow-up. In one patient, the PPK became progressive after a stable phase of 6 years, despite stable hematological findings (#12 in Table 3).

## 4. Discussion

In our prospective observational cross-sectional clinical study we found a rather positive influence of systemic chemotherapy on PPK, either in patients suffering from MM or from its precancerous stages—SMM and MGUS. In the group of ten MM patients receiving on-label systemic therapy, PPK improved in 30% and remained stable in the rest of the patients in the follow-up of up to 4.5 years. However, all patients in this group presented rather discrete corneal opacities. None of them required surgical treatment. In patients without systemic therapy, PPK stayed stable. In the literature, only a few case descriptions exist which report on the potential influence of systemic therapy directed against the underlying hematological malignancy on corneal findings associated with the respective hematological disorder. Prospective studies have not been available so far. An improvement of clinical and confocal microscopic findings of the cornea with the following improvement of vision was reported by Buerk et al. 6 months after chemotherapy in a case of PPK by MM [11] and by Houben et al. 4 months after chemotherapy and stem cell transplantation in a case of PPK by MM [15]. In a case published by Duquesne et al., early diagnosis and intensive treatment with autologous stem cell transplantation (autoSCT) in a case of MGUS with kidney and cornea involvement resulted in complete remission of PPK [5]. However, in all these cases, systemic treatment was performed in-label due to the advanced stage of the hematological disease, with consecutive therapy indications. These findings are consistent with our results regarding three MM patients who achieved complete PPK remission under systemic treatment. We confirmed these findings either under slit lamp or under IVCM.

In the group with precancerous stages of MG, three patients required PKP due to the massive deterioration of visual acuity associated with PPK. These patients underwent an off-label chemotherapy prior to the planned PKP to postpone or to avoid a quick accumulation of paraprotein in the graft. We hypothesized that the reduction of paraprotein levels in serum and remission of the underlying disease would help prevent PPK recurrence in the graft. In the postoperative follow-up period of up to two years, all these grafts remained clear. We observed discrete signs of PPK recurrence in one eye (#3, Table 3) after 27 months, without a deterioration of visual acuity so far. We therefore cannot exclude PPK recurrence in the future. However, in most cases in the literature and in our own experience (patient #1 in Table 3), PPK recurrence occurs within 12 months after PKP in patients without systemic treatment. Steinberg et al. suggested in 2011 that PKP was the treatment of choice [26]. Milman et al. reported in 2015 on the recurrence of PPK in three out of four patients after PKP and on visual rehabilitation with a reduction of corneal deposits in one case with SMM after chemotherapy and autoSCT [4]. In 2019, Skalicka et al. reported recurrent corneal opacification 9 months after PKP in a 61-year-old female patient with granular MGUS-associated PPK. In this patient, systemic chemotherapy was therefore started shortly after PKP of the second eye to reduce the risk of recurrence. Despite this treatment, progressive opacification of the first eye and the onset of opacification in the contralateral corneal graft occurred after 3 years. However, after repeated PKP in the first eye, and without additional systemic treatment, no further recurrence occurred in the following 5 years [17]. Since corneal deposits have been found to recur in patients after PKP [11,16,17,18,19,20,21,22,23,24], it seems prudent to postpone corneal transplantation until control of systemic disease is achieved [11]. Furthermore, we recommend a hematological check for appropriate diagnosis and treatment before making a decision on PKP for corneal opacities of unclear etiology.

The effect of systemic therapy on ocular findings has not been largely studied so far. Furthermore, ocular involvement in MG is not considered end-organ damage and is not yet considered an indication for systemic therapy [13]. However, according to the medical literature, systemic treatments with chemotherapeutic agents do not necessarily result in corneal clearing in advanced PPK, and deposits may recur in the corneal graft even after PKP [16,19,27]. This corresponds to the follow-up of four patients (#4, 5, 6 and 7) from the MM group (Table 2) in our study, whose PPK did not improve despite effective systemic treatment. A progression of PPK despite a concomitant reduction of IgG levels in serum using systemic therapy has also been reported [6]. The recently published retrospective study of MGOS showed a very high rate of PPK recurrence after PKP (4 out of 4 patients) and after systemic therapy (3 out of 12 patients) and no response of the MGOS after systemic therapy in 9 out of 12 patients [28].

The limitations of our study are the small number of cases, the difficulty of histopathological examination in non-surgical cases, and the lack of guidelines for surgical and conservative therapy of PPK. More studies in different populations will be necessary considering that these are pathologies whose manifestations and therapeutic responses can be modified by multiple factors, including differences in the population due to genetic aspects of this type of hematologic pathology.

To conclude, we conducted the first study prospectively evaluating the course of PPK associated with different stages of monoclonal gammopathy. We followed-up patients with and without systemic therapy as well as after PKP. We found a regression or stability of corneal findings in the MM group under in-label systemic therapy directed against the underlying hematological disease in order to reduce the paraprotein-secreting plasma cell clone. This supports the conclusion that clone reduction enables endogenous clearance mechanisms in the cornea to occur and, as a result, stabilizes visual acuity with reduced secondary production of paraproteins. Furthermore, we observed transparent corneal grafts without signs of PPK recurrence for up to two years in patients who received off-label systemic treatment (in non-MM cases) prior to planned PKP. Although we described only a few cases, we provided evidence for the positive influence of systemic hematological therapy on the translucency of corneal grafts in patients suffering from monoclonal gammopathy. Hence, we recommend performing systemic treatment to reduce serum paraprotein levels before planned PKP. The indication for off-label chemotherapy should always be discussed in an interdisciplinary fashion between ophthalmology and hematology specialists.

## Figures and Tables

**Table 1:** Demographic characteristics and hematological diagnosis of study participants. [SD = standard deviation, MM = multiple myeloma, MGUS = monoclonal gammopathy of undetermined significance, PPK = paraproteinemic keratopathy].

**PPK**	*n* = 22	**mean age ± SD**	**gender**	***n* =**	**mean age ± SD**
		68 ± 10.4	male	11	70.8 ± 11.4
			female	11	65.2 ± 9.4
		**hematological diagnosis**		***n* =**	**Systemic treatment *n* =**
		MM		10	10
		MGUS or lymphoma		12	3

**Table 2:** Ophthalmological and hematological follow-up of PPK patients requiring systemic treatment due to MM. First section (1–3): patients lost to follow-up due to death after 13–33 months; second section (4–7): stable PPK; third section (8–10): remission of PPK.

	Type of MM (Gender)	Form of PPK	BCVA (LogMAR) OD/OS	M-Protein (g/L)	Treatment (Response)	F o l l o w u p			
						Hematological	PPK	BCVA OD/OS	M-Protein (g/L)
**1**	IgG κ (f)	stromal flake-like opacity	0.5/0.5	9.6	VRd (PR), DRd (PR), autoSCT (PR)	death after 13 months	Lost in follow-up due to death. No change in PPK findings under slit lamp and IVCM until death	0.6/0.3	6.2
**2**	LC κ (m)	peripheral superficial band-like PPK	0.1/0.2	0.80	Vd (PR), Md (SD), Dd (VGPR)	death after 33 months		0.2/0.4	negative
**3**	LC λ (f)	stromal flake-like opacity	0.0/0.0	negative	VCd (VGPR), autoSCT (CR), after 1 year DRd for recurrence	death after 33 months		n/a	negative
**4**	IgA λ (f)	central golden-brown discoloration of the pre-descemet layer	0.1/0.8	50.0	VRd (VGPR) autoSCT (VGPR) VRd (CR)	CR for 4 years, no recurrence without therapy	no change in PPK findings under slit lamp and IVCM	0.0/0.1	7.7
**5**	LC λ with plasmacytoma in the bladder and intestine (m)	stromal flake-like opacity	0.2/0.1	negative	VCd (PR) cystoprostatectomy and pelvic lymphadenectomy with sigmoidectomy (CR)	CR, no recurrence up to 4 years		0.1/0.0	negative
**6**	IgA κ (m)	stromal flake-like opacity	0.2/0.2	negative	VCd (CR)	CR, watch and wait up to 4.5 years		0.1/0.1	negative
**7**	IgA λ (m)	stromal flake-like opacity	0.0/0.0	0.50	Vd (PR), DVd (PR) EPd (CR), autoSCT (CR)	CR, no recurrence up to 4 years		0.0/0.0	negative
**8**	IgA λ (f)	stromal flake-like opacity (Figure 1)	0.0/0.0	negative	VCd (VGPR), autoSCT (CR), for recurrence after 3 years: DKd (CR)	stringent CR no recurrence up to 2 years	complete recovery of PPK under slit lamp and IVCM	0.0/0.0	negative
**9**	IgG κ (f)	stromal punctiform crystalline-like PPK (Figure 2)	0.0/0.0	42.60	RVd (VGPR), autoSCT (CR)	CR no recurrence up to 3.5 years		0.0/0.0	negative
**10**	IgA λ (f)	stromal flake-like opacity, (Figure 3) a PPM in the form of focal detachment of the neurosensory retina	0.3/0.1	30.90	-DRd (stopped after 17 cycles due to progression), -IsaPd (VGPR)	Progression after therapy stopping	complete recovery of PPK under slit lamp and IVCM with temporary recovery of PPM under DRd, then recurrence of PPM after 14 months	0.1/0.1	50.9
								0.5/0.1	

1–10 = patient number, PPK = paraproteinemic keratopathy, Ig = immunoglobulin, κ = kappa, λ = lambda, LC = light chain, IVCM = in vivo laser scanning confocal microscopy, PR = partial remission, VGPR = very good partial remission, CR = complete remission, SD = stable disease, VRd = bortezomib/lenalidomide/dexamethasone, VCd = bortezomib/cyclophosphamide/dexamethasone, DVD = daratumumab/bortezomib/dexamethasone, Vd = bortezomib/dexamethasone, Md = melphalan/dexamethasone, DRd = daratumumab/lenalidomide/dexamethasone, EPd = elotuzumab plus/pomalidomide/dexamethasone, DKd = daratumomab/cafilzomib/dexamethasone, IsaPd = isatuximab/pomalidomide/dexamethasone, Dd = daratumumab/dexamethasone, autoSCT = autologous stem cell transplantation, PPM = paraproteinemic maculopathy, m = male, f = female.

**Table 3:** Ophthalmological and hematological follow-up of PPK patients without hematological indication for treatment. First section: patients after PKP without systemic treatment; second section: patients after PKP with systemic treatment; third section: patients without PKP and without systemic treatment.

	Hematological Diagnosis (Gender)	Form of PPK	BCVA (LogMar) OD/OS	M-Protein (g/L)	Treatment (Response)	F o l l o w u p			
						Hematological	PPK (Term)	BCVA (LogMar) OD/OS	M-Protein (g/L)
**1**	MGUS of type IgG κ (m)	stromal flake-like opacity (Figure 4)	0.6/0.2	8	no systemic treatment	SD	OD/OS: recurrence of PPK in the grafts within 12 months after PKP, then stable up to 6 years	0.6/0.3	8.4
					PKP in both eyes				
**2**	Systemic (AL) amyloidosis type lambda (m)	deep stromal crystalline PPK (Figure 5)	0.5/1.3	44	VRd (VGPR)	watch and wait in VGPR up to 20 months	OD: no recurrence in the graft up to 20 months after PKP	0.5/1.3	16.3
					PKP in one eye (OD)				
**3**	lymphoplasmacytoid immunocytoma with MGUS of type IgG κ (m)	stromal flake-like opacity (Figure 6)	0.2/0.5	26.4	1- rituximab-dexamethasone (SD) 2- rituximab-bendamustine (PR)	improved remission after the surgery by weekly maintenance therapy with Vd	OD: progression of PPK and BCVA OS: no recurrence in the graft for up to 24 months after PKP After 27 months: very delicate opacities with stable BCVA for up to 36 months	0.5/0.0	10.6
					PKP in one eye (OS) by PR			0.7/0.0	4.5
**4**	MGUS of type IgG κ (f)	stromal punctiform crystalline-like PPK (Figure 7)	0.4/0.2	18.6	DRd (PR)	watch and wait	OD: no recurrence for up to 18 months after PKP OS: stable	0.4/0.2	4.1
					PKP in one eye (OD)				
**5**	MGUS of type IgG κ (*n* = 3) (f, m, f)	stromal flake-like opacity (*n* = 3)	0.0/0.0	3.50	no systemic treatment and no eye treatment	SD	stable findings under slit lamp and IVCM	0.0/0.0	4.5
**6**			0.0/0.0	0.40		SD	stable findings under slit lamp and IVCM	0.0/0.0	n/a
**7**			0.0/0.0	15.20		SD, watch and wait	stable findings under slit lamp and IVCM	0,0/0.0	22.5
**8**	MGUS of type IgG λ (f)	stromal flake-like opacity	0.3/0.0	negative		SD	stable findings under slit lamp and IVCM	0.3/0,0	1.8
**9**	MGUS of type IgA κ (m)	stromal flake-like opacity	0.8/0.1	negative		SD	stable findings under slit lamp and IVCM	0.6/0.2	negative
**10**	MGUS of type LC κ (m)	stromal lattice-like PPK	0.2/0.5	negative		SD	stable findings under slit lamp and IVCM	0.3/0.6	negative
**11**	MGUS of type LC λ (m)	stromal punctiform crystalline-like PPK	0.2/0.2	n/a		SD	stable findings under slit lamp and IVCM	0.3/0.3	n/a
**12**	SMM of type IgA λ (f)	peripheral superficial band-like PPK	0.0/0.0	0.50		SD	Increase in opacities and thinning of peripheral cornea with decrease in BCVA after 6 years	0.2/0.1	0.3

1–12 = patient number, PPK = paraproteinemic keratopathy, Ig = immunoglobulin, κ = kappa, λ = lambda, LC = light chain, IVCM = in vivo laser scanning confocal microscopy, PR = partial remission, VGPR = Very good partial remission, CR = Complete remission, SD = Stable disease, VRd = Bortezomib (Velcade^®^, Janssen-Cilag International NV, Olen, Belgium)/Lenalidomid/dexamethasone, Vd = Bortezomib/dexamethasone, DRd = daratumumab/lenalidomide/dexamethasone, PKP = penetrating keratoplasty, n/a = not available, BCVA = best-corrected visual acuity (in LogMAR), OD = oculus dexter, OS = oculus sinister, m = male, f = female.

## Data Availability

The study data is stored in the archives of the Department of Ophthalmology, University Medical Center of the Johannes Gutenberg University Mainz, Mainz, Germany.
